# Supplementary figures and images for: VGLL2 and TEAD1 fusion proteins drive YAP/TAZ-independent tumorigenesis by engaging p300
Source: bioRxiv. 2025 Feb 18:2024.05.01.592016. Originally published 2024 May 3. Preprint. [Version 2] doi: 10.1101/2024.05.01.592016 (PMC11092657; doi:10.1101/2024.05.01.592016)

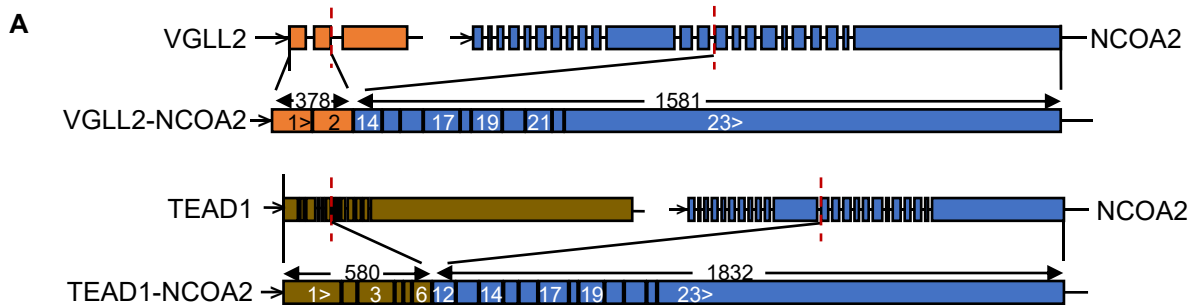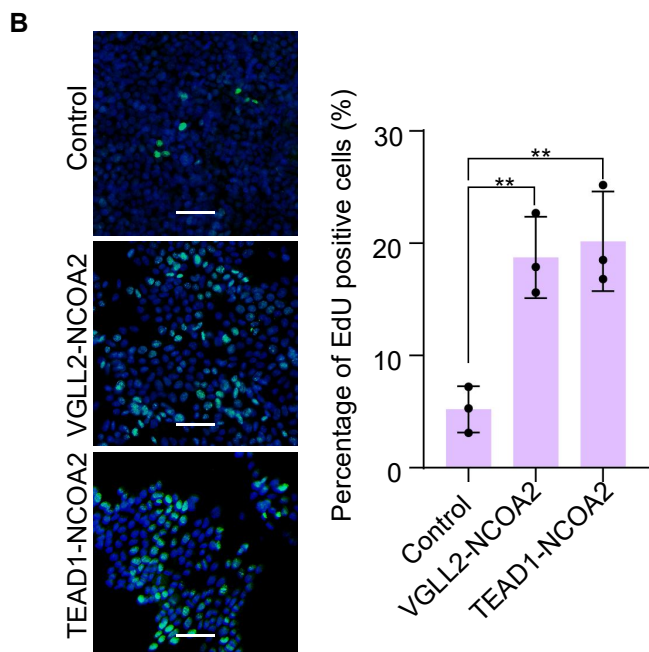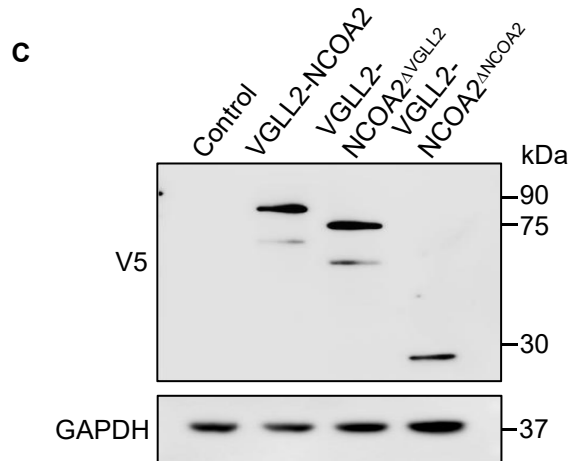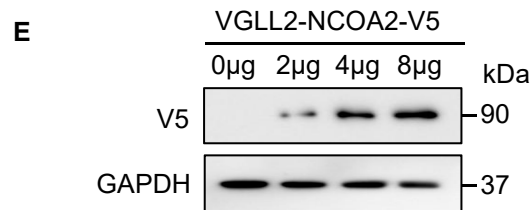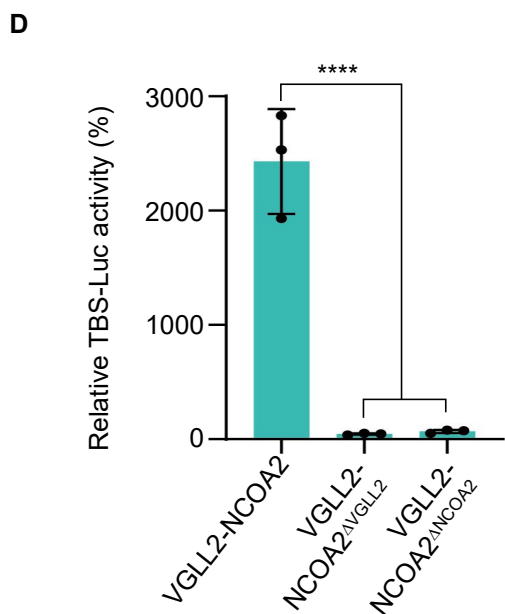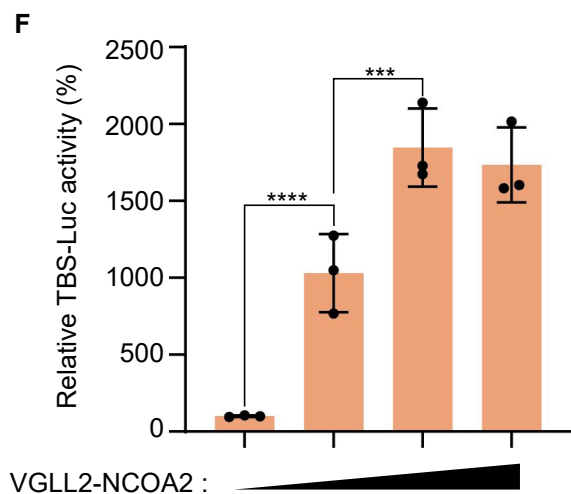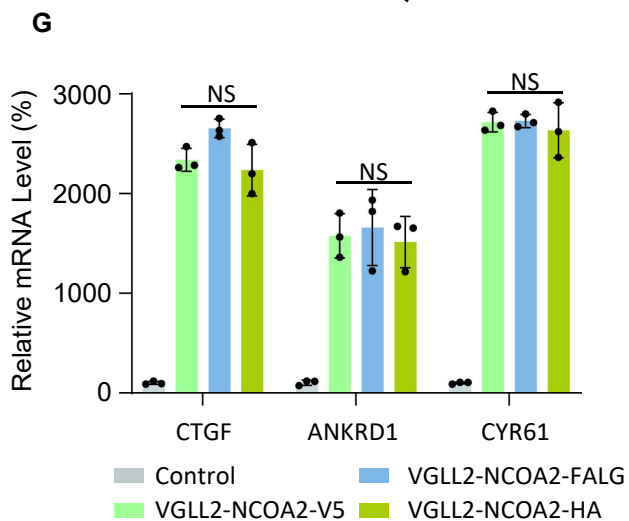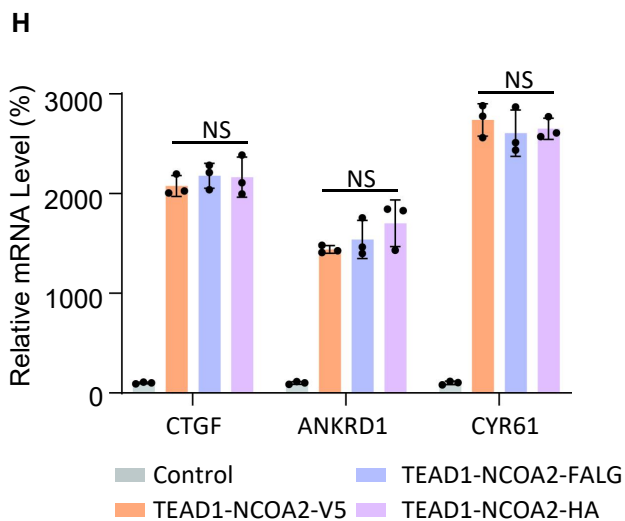

Supplement: Supplement 1 — Figure 1-figure supplement 1: VGLL2-NCOA2 regulates TEAD-dependent reporter activity. A. Schematic representation of the exons and breaking points of the VGLL2, TEAD1 and NCOA2 genes involved in generating VGLL2::NCOA2 and TEAD1::NCOA2 gene arrangement. B. EdU staining showing cell proliferation of HEK293T cells transfected with VGLL2-NCOA2 or TEAD1-NCOA2. Bar chart showing the percentage of EdU-positive cells. Scale bars, 200 μm, n=3; **, p < 0.01. C. Immunoblot analysis of VGLL2-NCOA2, VGLL2-NCOA2ΔVGLL2 and VGLL2-NCOA2ΔNCOA2 in HEK293T cells transfected with the expression constructs carrying the V5 tag. D. TBS-Luc reporter activity in HEK293T cells expressing VGLL2-NCOA2, VGLL2--NCOA2ΔVGLL2, and VGLL2-NCOA2ΔNCOA2. n = 3. ****, p < 0.0001. E. Immunoblot analysis of VGLL2-NCOA2-V5 in HEK293T cells transfected with different amount of expression constructs. F. VGLL2-NCOA2 promotes the activation of TBS-Luc reporter in dose-dependent manner. n = 3. ***, p < 0.001; ****, p < 0.0001. G-H. Relative mRNA levels of CTGF, ANKRD1, and CYR61 in HEK293T cells expressing VGLL2-NCOA2 (G) or TEAD1-NCOA2 (H) with HA (5’ end), FLAG (3’ end) or V5 (3’ end) tag. n=3; NS, no significance. [file media-1.pdf]

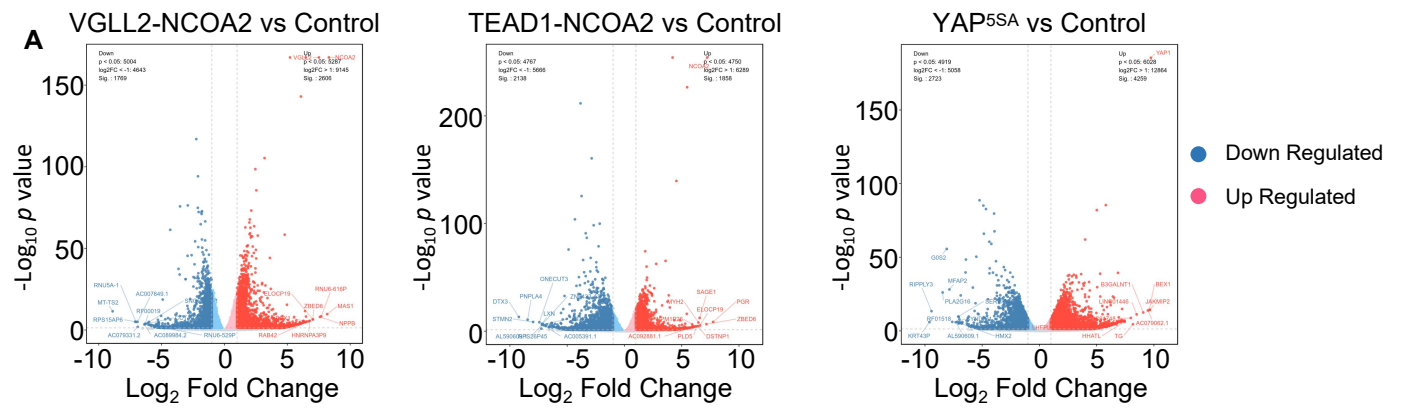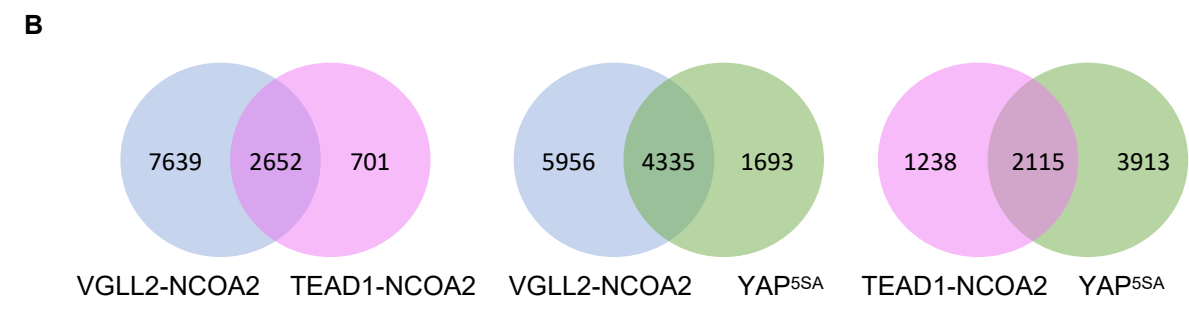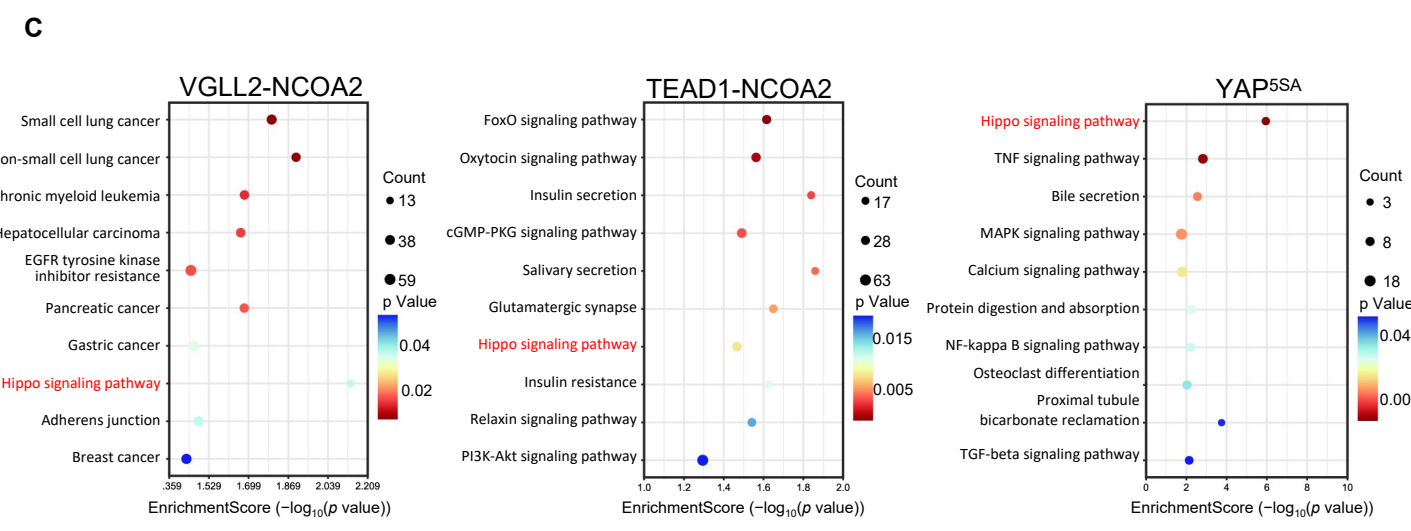

Supplement: Supplement 2 — Figure 3-figure supplement 1: Analysis of VGLL2-NCOA2, TEAD1-NCOA2 and YAP5SA-induced transcriptomes. A. Volcano maps of RNA-seq data sets of HEK293T cells expressing VGLL2-NCOA2, TEAD1-NCOA2, or YAP5SA. Red dots represent upregulated mRNAs. Blue dots represent downregulated mRNAs. p < 0.05, Log2FoldChange > 1 or < −1. B. Veen diagram showing the overlaps of differentially regulated genes identified by RNA-seq in HEK293T cells expressing VGLL2-NCOA2, TEAD1-NCOA2, or YAP5SA. C. KEGG pathway enrichment analysis of differentially regulated genes identified by RNA-seq in HEK293T cells expressing VGLL2-NCOA2, TEAD1-NCOA2 or YAP5SA. “Hippo signaling pathway” is highlighted in red. [file media-2.pdf]

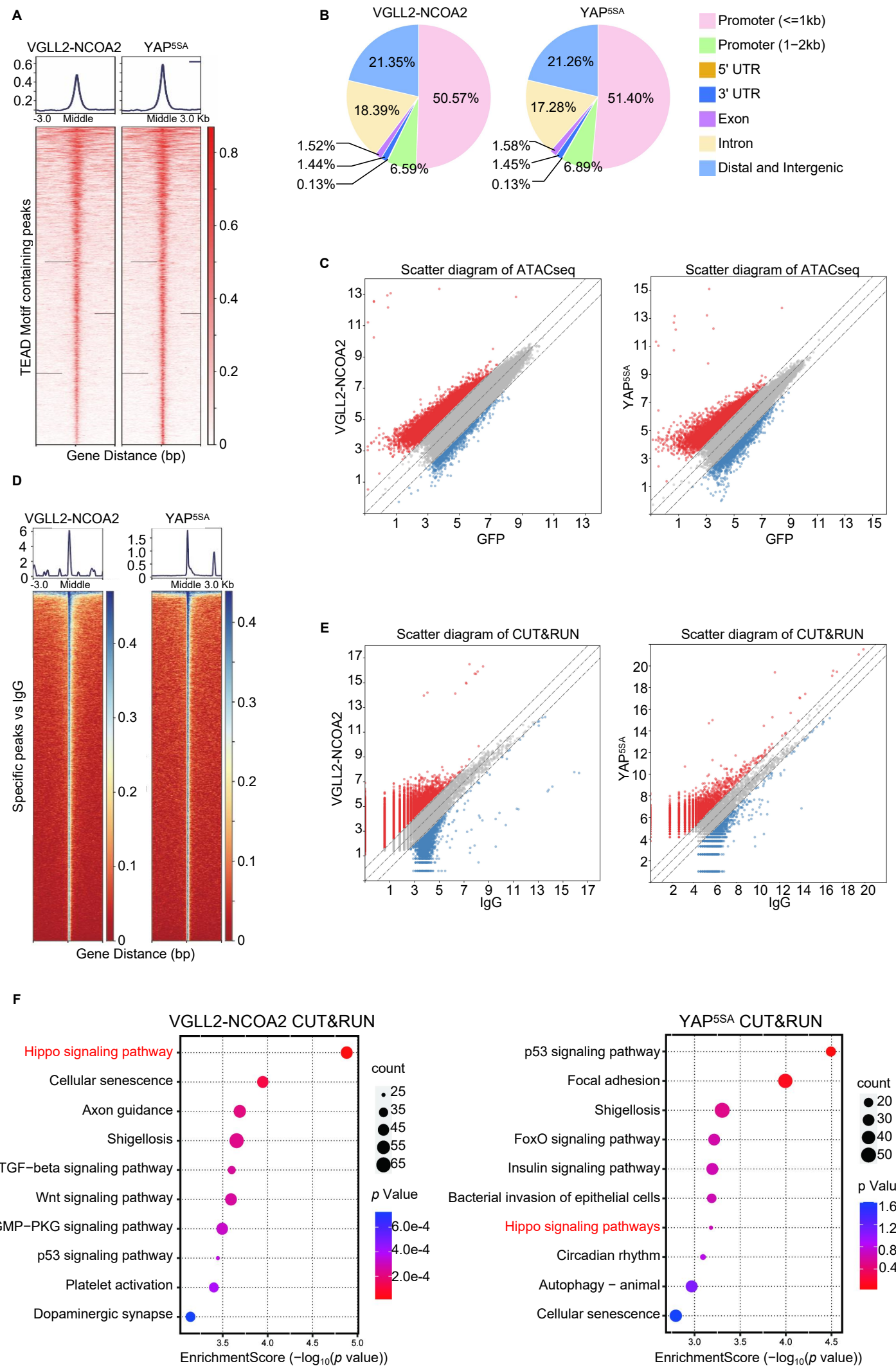

Supplement: Supplement 3 — Figure 3-figure supplement 2: ATAC-seq and CUT&RUN data characterization in VGLL2-NCOA2 and YAP5SA-expressing cells. A. Heatmaps of TEAD-motif containing ATAC-seq peaks in HEK293T cells expressing VGLL2-NCOA2 or YAP5SA. B. Distribution of ATAC-seq peaks in HEK293T cells expressing VGLL2-NCOA2 or YAP5SA. For VGLL2-NCOA2 ATAC-seq peaks, Promoter (<=1kb) 50.57%, Promoter (1−2kb) 6.59%, 5’ UTR 0.13%, 3’ UTR 1.44%, Exon 1.52%, Intron 18.39%, and Distal and Intergenic 21.35%. For YAP5SA ATAC-seq peaks, Promoter (<=1kb) 51.4%, Promoter (1−2kb) 6.89%, 5’ UTR 0.13%, 3’ UTR 1.45%, Exon 1.58%, Intron 17.28%, and Distal and Intergenic 21.26%. n=2. C. VGLL2-NCOA2 promotes chromatin accessibility. Scatter diagrams of ATAC-seq peaks show more up-regulated (red) than downregulated (blue) in HEK293T cells transfected with VGLL2-NCOA2 or YAP5SA. D. Heatmaps of VGLL2-NCOA2 and YAP5SA CUT&RUN peaks. E. VGLL2-NCOA2 and YAP genomic occupancy. Scatter diagrams showing more up-regulated CUT&RUN peaks (red) than down-regulated CUT&RUN peaks (blue) of VGLL2-NCOA2 and YAP5SA. F. KEGG pathway enrichment analysis of VGLL2-NCOA2 and YAP5SA CUT&RUN peaks. “Hippo signaling pathway” is highlighted in red. [file media-3.pdf]
