# Supplementary material for: VGLL2 and TEAD1 fusion proteins drive YAP/TAZ-independent transcription and tumorigenesis by engaging p300
Source: bioRxiv. 2024 May 3:2024.05.01.592016. Preprint. [Version 1] doi: 10.1101/2024.05.01.592016 (PMC11092657; doi:10.1101/2024.05.01.592016)
Supplement: Supplementary file 1 [file NIHPP2024.05.01.592016v1-supplement-1.pdf]

bioRxiv preprint doi: https://doi.org/10.1101/2024.05.01.592016; this version posted May 3, 2024. The copyright holder for this preprint (which was not certified by peer review) is the author/funder, who has granted bioRxiv a license to display the preprint in perpetuity. It is made available under aCC-BY 4.0 International license.

Supplement figure legends

Supplementary Figure 1: VGLL2-NCOA2 regulates TEAD-dependent reporter activity.

A. Schematic representation of protein structure of VGLL2-NCOA2, VGLL2-NCOA2 ΔVGLL2, and VGLL2-NCOA2 ΔNCOA2. Tondu motif (TDU) and transcriptional activation domain (TAD).

B. TBS-Luc reporter activity in HEK293T cells expressing VGLL2-NCOA2, VGLL2-NCOA2 ΔVGLL2, and VGLL2-NCOA2 ΔNCOA2. n = 3. ****, $p < 0.0001$.

C. Immunoblot analysis of VGLL2-NCOA2, VGLL2-NCOA2 ΔVGLL2 and VGLL2-NCOA2 ΔNCOA2 in HEK293T cells transfected with the expression constructs carrying the V5 tag.

D. VGLL2-NCOA2 promotes the activation of TBS-Luc reporter in dose-dependent manner. n = 3. ***, $p < 0.001$; ****, $p < 0.0001$.

E. Immunoblot analysis of VGLL2-NCOA2-V5 in HEK293T cells transfected with different amount of expression constructs.

Supplementary Figure 2: Analysis of VGLL2-NCOA2, TEAD1-NCOA2 and YAP^{5SA}-induced transcriptomes.

A.Volcano maps of RNA-seq data sets of HEK293T cells expressing VGLL2-NCOA2, TEAD1-NCOA2 or YAP^{5SA}. Red dots represent upregulated mRNAs. Blue dots represent downregulated mRNAs. $p < 0.05$, Log_2FoldChange > 1 or < -1.

B.Veen diagram showing the overlaps of differentially regulated genes identified by RNA-seq in HEK293T cells expressing VGLL2-NCOA2, TEAD1-NCOA2 or YAP^{5SA}.

C. KEGG pathway enrichment analysis of differentially regulated genes identified by RNA-seq in HEK293T cells expressing VGLL2-NCOA2, TEAD1-NCOA2 or YAP^{5SA}. "Hippo signaling pathway" highlighted in red.

Supplementary Figure 3: Characterization of ATAC-seq and CUT&RUN data in VGLL2-NCOA2 and YAP^{5SA}-expressing cells.

A. Heatmaps of TEAD-motif containing ATAC-seq peaks in HEK293T cells expressing VGLL2-NCOA2 or YAP^{5SA}.

B. Distribution of ATAC-seq peaks in HEK293T cells expressing VGLL2-NCOA2 or YAP^{5SA}. For VGLL2-NCOA2 ATAC-seq peaks, Promoter (<=1kb) 50.57%, Promoter (1–2kb) 6.59%, 5' UTR 0.13%, 3' UTR 1.44%, Exon 1.52%, Intron 18.39%, and Distal and Intergenic 21.35%. For YAP^{5SA} ATAC-seq peaks, Promoter (<=1kb) 51.4%, Promoter (1–2kb) 6.89%, 5' UTR 0.13%, 3' UTR 1.45%, Exon 1.58%, Intron 17.28%, and Distal and Intergenic 21.26%.

C. VGLL2-NCOA2 promotes chromatin accessibility. Scatter diagrams of ATAC-seq peaks showing more up-regulated peaks (red) than downregulated peaks (blue) in HEK293T cells transfected with VGLL2-NCOA2 or YAP^{5SA}.

D. Heatmaps of VGLL2-NCOA2 and YAP^{5SA} CUT&RUN peaks.

E. VGLL2-NCOA2 and YAP genomic occupancy. Scatter diagrams showing more up-regulated CUT&RUN peaks (red) than down-regulated CUT&RUN peaks (blue) of VGLL2-NCOA2 and YAP^{5SA}.

F. KEGG pathway enrichment analysis of VGLL2-NCOA2 and YAP^{5SA} CUT&RUN peaks. "Hippo signaling pathway" highlighted in red.

bioRxiv preprint doi: https://doi.org/10.1101/2024.05.01.592016; this version posted May 3, 2024. The copyright holder for this preprint (which was not certified by peer review) is the author/funder, who has granted bioRxiv a license to display the preprint in perpetuity. It is made available under aCC-BY 4.0 International license.

Supplementary Figure 1

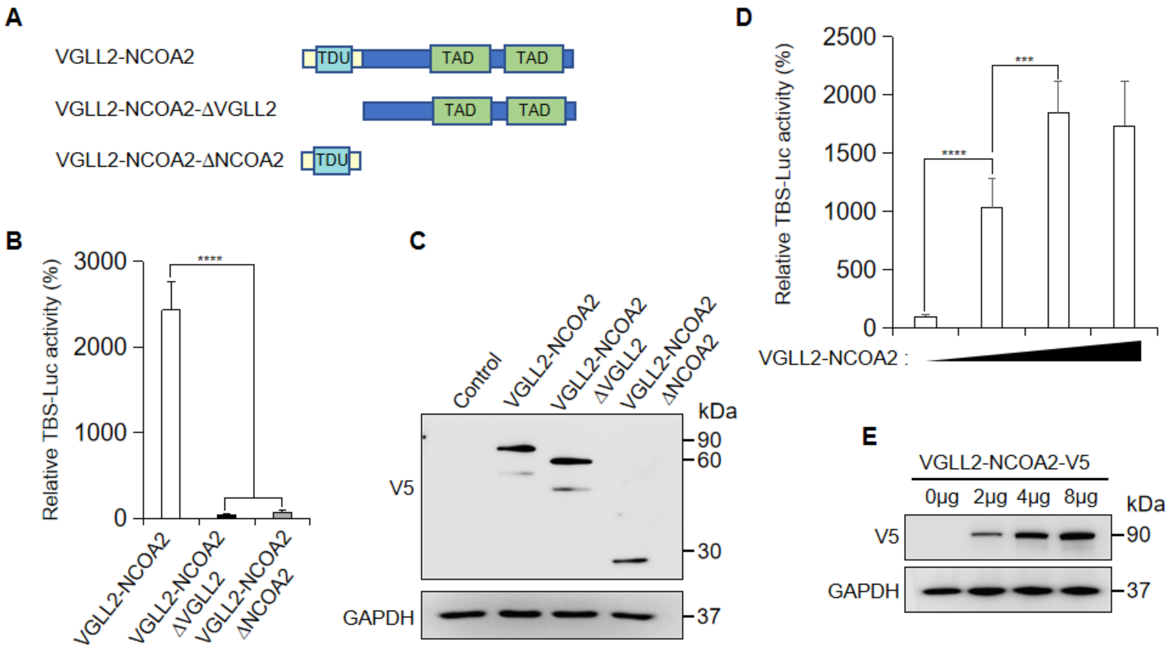

bioRxiv preprint doi: https://doi.org/10.1101/2024.05.01.592016; this version posted May 3, 2024. The copyright holder for this preprint (which was not certified by peer review) is the author/funder, who has granted bioRxiv a license to display the preprint in perpetuity. It is made available under aCC-BY 4.0 International license.

Supplementary Figure 2

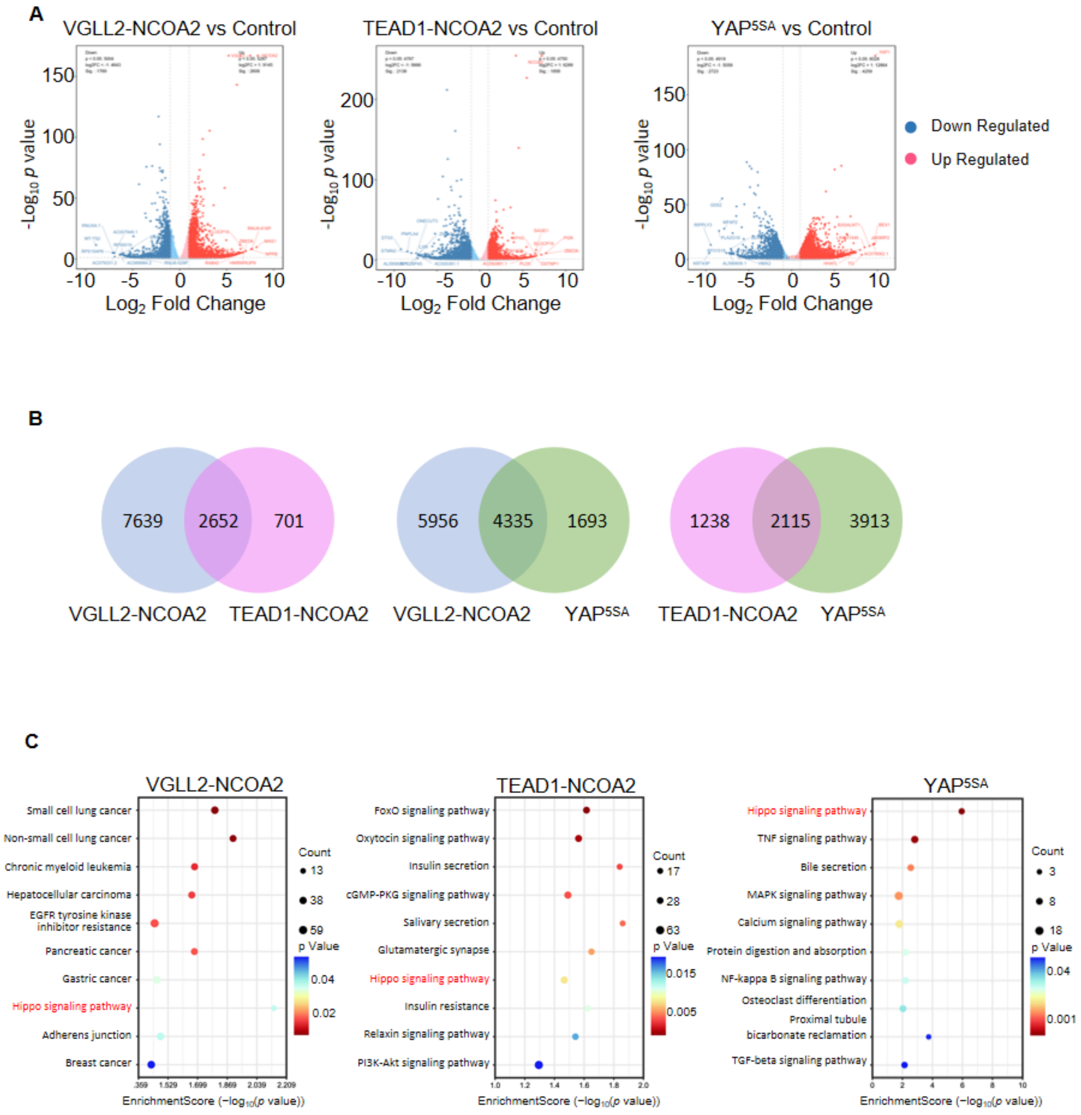

bioRxiv preprint doi: https://doi.org/10.1101/2024.05.01.592016; this version posted May 3, 2024. The copyright holder for this preprint (which was not certified by peer review) is the author/funder, who has granted bioRxiv a license to display the preprint in perpetuity. It is made available under aCC-BY 4.0 International license.

Supplementary Figure 3

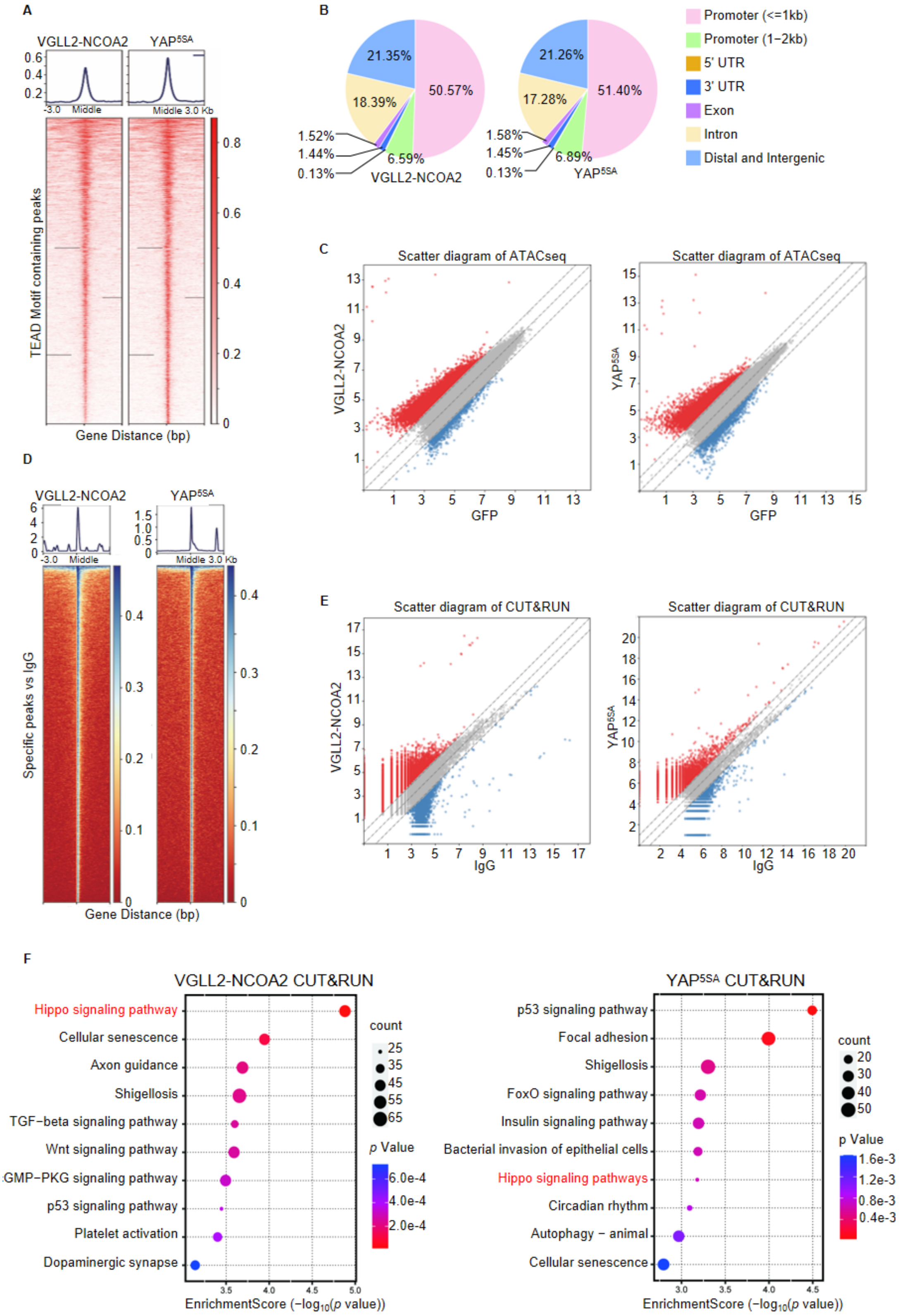

bioRxiv preprint doi: https://doi.org/10.1101/2024.05.01.592016; this version posted May 3, 2024. The copyright holder for this preprint (which was not certified by peer review) is the author/funder, who has granted bioRxiv a license to display the preprint in perpetuity. It is made available under aCC-BY 4.0 International license.

Supplementary Table 5: Primers used for qPCR

Name	Primer
GAPDH-F'	GGAGCGAGATCCCTCCAAAAT
GAPDH-R'	GGCTGTTGTCATACTTCTCATGG
CTGF-F'	CAGCATGGACGTTCGTCTG
CTGF-R'	AACCACGGTTTGGTCCTTGG
ANKRD1-F'	GCCTACGTTTCTGAAGGCTG
ANKRD1-R'	GTGGATTCAAGCATATCACGGAA
CYR61-F'	CAGGACTGTGAAGATGCGGT
CYR61-R'	GCCTGTAGAAGGGAAACGCT
Actb-F'	GTGACGTTGACATCCGTAAAGA
Actb-R'	GCCGGACTCATCGTACTCC
Ctgf-F'	GACCCAACTATGATGCGAGCC
Ctgf-R'	CCCATCCCACAGGTCTTAGAAC
Ankrd1-F'	GGATGTGCCGAGGTTTCTGAA
Ankrd1-R'	GTCCGTTTATACTCATCGCAGAC
Cyr61-F'	TAAGGTCTGCGCTAAACAACTC
Cyr61-R'	CAGATCCCTTTCAGAGCGGT
